# The complete chloroplast genome sequence of *Actinidia Rufa* (Actinidiaceae)

**DOI:** 10.1080/23802359.2018.1450676

**Published:** 2018-05-09

**Authors:** Sang-Chul Kim, Jei-Wan Lee, Seung-Hoon Baek, Min-Woo Lee, Young-Je Kang

**Affiliations:** a Division of Forest Genetic Resources, National Institute of Forest Science, Suwon-si, Korea;; b Warm-temperature and subtropical Forest Research center, National Institute of Forest Science, Suwon-si, Korea

**Keywords:** Chloroplast, *Actinidia rufa*, genome sequence, Actinidiaceae

## Abstract

The complete chloroplast (cp) genome sequence of *Actinidia rufa* was determined by next-generation sequencing in this study. The whole cp genome was 156,543 bp in length, containing a large single-copy (LSC) of 88,435 bp and a small single-copy (SSC) region of 20,307 bp, which was separated by a pair of 23,900 bp inverted repeat (IR) regions. The genome contained 131 genes, including 84 protein-coding genes, 39 tRNA genes, 8 ribosomal RNA genes. Two events were found in the Actinidiaceae chloroplast genome. One was the deletion of the *clpP* gene and the other was the *trnfM*-CAU gene in the same direction in LSC region. The phylogenetic position of *A. rufa* was closely clustered with *A. chinensis*, *A. deliciosa* as sister species.

Kiwifruit is an important fruit tree in the Actinidiaceae. It is rich in vitamins C and P, and contains trace elements and a variety of amino acids and enzymes that the human body needs (Lan et al. 2017). This crop plant is cultivated in many countries. However, bacteria, caused by *Pseudomonas syringae* pv. *actinidiae* (Psa), is currently the major cause of losses in kiwifruit production worldwide (Yao et al. 2015). The *Actinidia* consists of about 55 species distributed in East and South Asia. The *A. rufa* (Siebold and Zucc.) Planch. ex Miq. is native to subtropical and warm-temperate regions, including Korea, Taiwan, and Japan. In this study, we report the first complete chloroplast genome of *A. rufa* based on the next-generation sequencing data and compared it with those of 8 other species genomes available from the Actinidiaceae.

A wild individual of *A. rufa* was sampled from seashore in Cheongsu-ri, Hangyeong-myeon, Jeju-si, Jeju-do, Republic of Korea (N: 33° 18′16″, E: 126° 15′ 57″). Its genomic DNA was isolated from the fresh leaves with a Plasmid SV mini kit (GeneAll Biotechnology, Seoul, Korea) and stored in a DNA bank in the Forest Genetic Resources Department (NIFS_0122063630). The whole genome sequencing was conducted on the Ion torrent Platform (Life Technologies, Carlsbad, CA, USA). The filtered sequences were assembled with reference sequence of *A. polygama* (Genbank: NC031186). The sequenced fragments were assembled using Geneious 10.2.3 (Biomatters, Auckland, New Zealand; Kearse et al. 2012). Annotation was performed using the BLAST searches. All the tRNA sequences were confirmed using the web-based online tool, tRNAScan-SE (Schattner et al. 2005) with default settings to corroborate tRNA boundaries identified by Geneious. The maximum likelihood (ML) tree searches and ML bootstrap searches were performed using the RAxML Blackbox web-server (http://phylobench.vital-it.ch/raxml-bb/, Stamatakis et al. 2008) from alignments created with MAFFT (Katoh et al. 2002) using plastid genomes of 9 species. The RAxML analyses were run with a rapid bootstrap analysis using a random starting tree and 100 maximum likelihood bootstrap replicates.

The complete cp genome of *A. rufa* was a double-stranded, circular DNA of 156,543 bp in length, which contains two inverted repeat (IR) regions of 23,900 bp each separated by large single-copy (LSC) and a small single-copy (SSC) region of 88,435 and 20,307 bp, respectively (NCBI acc. no. MF980719). The genome contained 131 genes, including 84 protein-coding genes, 39 tRNA genes, 8 ribosomal RNA genes. Most of these genes were single copy genes, The five protein-coding genes, eight tRNA genes and four rRNA genes were duplicated in IR region.

The phylogenetic tree was constructed with RAxML based on 9 complete chloroplast genome sequences of Actinidiaceae (Figure 1). The phylogenetic position of *A. rufa* was closely clustered with *A. chinensis*, *A. deliciosa* as sister species and then clustered with *A. eriantha* and *A. tetramera*. Two events were found in the Actinidiaceae chloroplast genome. One was the deletion of the *clpP* gene and the other was the duplication of *trnfM*-CAU gene in the same direction in LSC region.

**Figure 1.**
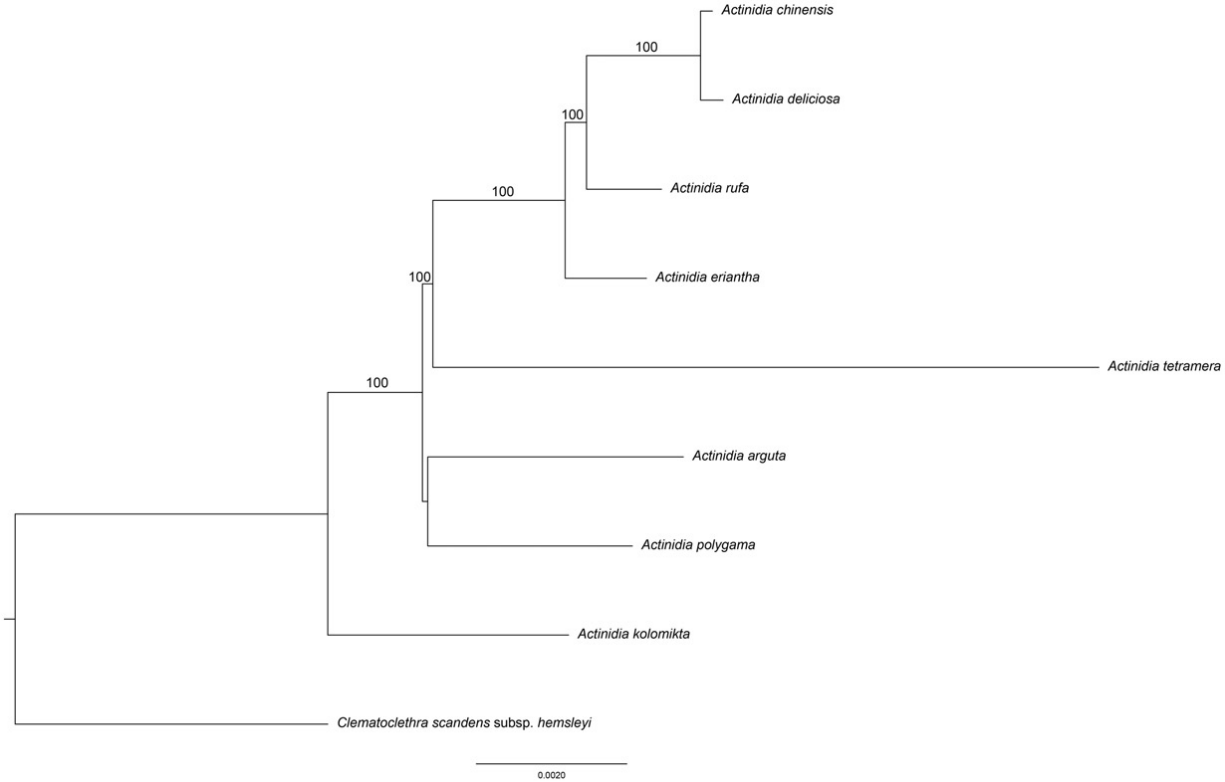
The phylogenetic tree based on the 9 complete chloroplast genome sequences. Bootstrap support values >50% are given at the nodes. Accession Numbers: *Actinidia chinensis* (NC026690), *A. deliciosa* (NC026691), *A. eriantha* (NC034914), *A. tetramera* (NC031187), *A. arguta* (NC034913)*, A. polygama* (NC031186), *A. kolomikta* (NC034915), *Clematoclethra scandens* subsp. Hemsleyi (KX345299).
